# Comparison of Multiple Bioactive Constituents in Different Parts of *Eucommia ulmoides* Based on UFLC-QTRAP-MS/MS Combined with PCA

**DOI:** 10.3390/molecules23030643

**Published:** 2018-03-13

**Authors:** Ying Yan, Hui Zhao, Cuihua Chen, Lisi Zou, Xunhong Liu, Chuan Chai, Chengcheng Wang, Jingjing Shi, Shuyu Chen

**Affiliations:** College of Pharmacy, Nanjing University of Chinese Medicine, Nanjing 210023, China; yanying93ly@163.com (Y.Y.); zhaohui_199301@163.com (H.Z.); cuihuachen2013@163.com (Cui.C.); zlstcm@126.com (L.Z.); echo_0523@hotmail.com (Chu.C.); ccw199192@163.com (C.W.); shijingjingquiet@163.com (J.S.); 18305172513@163.com (S.C.)

**Keywords:** *Eucommia ulmoides*, UFLC-QTRAP-MS/MS, multiple bioactive constituents, simultaneous determination, PCA

## Abstract

*Eucommia ulmoides* Oilv. (EU), also called Du-zhong, is a classical traditional Chinese medicine. Its bark, leaf, and male flower are all used for medicinal purposes, called Eucommiae Cortex (EC), Eucommiae Folium (EF), and Eucommiae Flos Male (EFM). In order to study the difference in synthesis and the accumulation of metabolites in different parts of EU, a reliable method based on ultra-fast liquid chromatography tandem triple quadrupole mass spectrometry (UFLC-QTRAP-MS/MS) was developed for the simultaneous determination of a total of 21 constituents, including two lignans, 6 iridoids, 6 penylpropanoids, 6 flavonoids, and one phenol in the samples (EC, EF, and EFM). Furthermore, principal component analysis (PCA) was performed to evaluate and classify the samples according to the contents of these 21 constituents. All of the results demonstrated that the chemical compositions in EC, EF, and EFM were significantly different and the differential constituents (i.e., aucubin, geniposidic acid, chlorogenic acid, pinoresinol-di-*O*-β-d-glucopyranoside, geniposide, cryptochlorogenic acid, rutin, and quercetin) were remarkably associated with sample classifications. The research will provide the basic information for revealing the laws of metabolite accumulation in EC, EF, and EFM from the same origin.

## 1. Introduction

Eucommiae Cortex (EC), Eucommiae Folium (EF), and Eucommiae Flos Male (EFM) are derived from the dried bark, leaf, and male flower of *Eucommia ulmoides* Oilv. (EU), respectively. EC has been used as an important traditional Chinese medicine (TCM) for more than 2000 years in China, and EF has become a popular functional health food and plant medicine material in past twenty years. EC and EF are officially documented in the Chinese Pharmacopoeia [1]. Though the studies about EFM started relatively late, accumulating evidence has proved that EFM, like EC and EF, was found to be rich in bioactive constituents [2,3,4].

Phytochemical investigations have revealed that EU mainly contains several types of constituents, such as lignans, iridoids, penylpropanoids, and flavonoids [5]. Pharmacological studies demonstrated that lignans have biological activities including anti-hypertensive, anti-tumor, anti-inflammatory, liver-protection, and inhibiting platelet activation; iridoids are known to have a variety of biological activities such as improvement in the collagen synthesis, anti-aging, anti-tumor, anti-obesity and immunity promotion, penylpropanoids possess antimicrobial, anti-inflammatory, anti-tumor, anti-virus, anti-oxidative, and other biological activities; there are also a great number of bioactive properties such as antiviral, anti-inflammatory, anti-oxidant, and glycation inhibitory existing in flavonoids [6,7,8,9,10,11,12]. Reports mentioned above indicated that these potentially bioactive constituents may be responsible for the various biological activities of EU. For example, the antimicrobial, anti-inflammatory, and analgesic effects of EFM are stronger than EC; EF may be associated with the difference of the content of flavonoids and polysaccharides in different parts of EU [13].

In recent years, various analytical methods have been reported as tools for EC quality assessment, including high-performance liquid chromatography (HPLC) [2,14,15], high-performance capillary electrophoresis (HPCE) [16], and ultra-performance liquid chromatography-tandem mass spectrometry (UPLC-MS) [17]. The ultra-fast performance liquid chromatography coupled with triple quadrupole-linear ion trap mass spectrometry (UFLC-QTRAP-MS/MS) method developed in this study is highly sensitive, strongly selective, quantitatively accurate, and results in fast analysis. Compared with the HPLC method, the developed method has the advantages of good separation efficiency, high peak capacity, and high sensitivity, which is more suitable for the separation and analysis of the complex systems of TCMs. UFLC-QTRAP-MS/MS is equipped with multiple reaction monitoring (MRM) for quantitative analysis, which is a data acquisition technology that possesses high sensitivity, precision, and throughput, and one transition each from the precursor to the reporter ions is utilized for every constituent, respectively [18,19]. Otherwise, not only the quadrupole linear ion trap system retains all of the traditional functions of the standard triple quadrupole, but also in the linear ion trap mode, the QTRAP system can improve the sensitivity of the full scan mode, enhance the ion scan, and provide the multiple ion scan function [20].

The aim of our experiments is to study the difference of synthesis and accumulation of metabolites in different parts of EU based on simultaneous determination of multiple bioactive constituents combined with PCA. A reliable method based on UFLC-QTRAP-MS/MS was developed for the simultaneous determination of 21 constituents, including two lignans, 6 iridoids, 6 penylpropanoids, 6 flavonoids, and one phenol contained in EU samples (EC, EF, and EFM). Furthermore, PCA was performed to evaluate and classify the samples according to the contents of these 21 constituents. To the best of our knowledge, this is the most comprehensive published report in the quantitative analysis of EU.

## 2. Materials and Methods

### 2.1. Chemicals and Reagents

Methanol and acetonitrile of HPLC grade were purchased from Merck (Damstadt, Germany). Ultrapure water was prepared using a Milli-Q purifying system (Millipore, Bedford, MA, USA). Other reagents were analytical grade (Sino Pharm Chemical Reagent Co., Ltd., Shanghai, China). The chemical structures of 21 constituents including catalpol (**1**), aucubin (**2**), harpagide (**3**), geniposidic acid (**4**), neochlorogenic acid (**5**), protocatechuic acid (**6**), syringin (**7**), chlorogenic acid (**8**), pinoresinol-di-*O*-β-d-glucopyranoside (**9**), geniposide (**10**), cryptochlorogenic acid (**11**), caffeic acid (**12**), rutin (**13**), isoquercitrin (**14**), genipin (**15**), pinoresinol-4′-*O*-β-d-glucopyranoside (**16**), astragalin (**17**), isochlorogenic acid A (**18**), kaempferol (**19**), baicalein (**20**), and quercetin (**21**) were shown in Figure S1. The reference substances of **2**, **3**, **4**, **7**, **8**, **9**, **10**, **12**, **13**, **17**, **19**, **20**, and **21** were purchased from the Chinese National Institute of Control of Pharmaceutical and Biologiceal Products (Beijing, China). The reference substances of **1** and **15** were purchased from Nanjing Boaoqi-Biotechnology Co., Ltd. (Nanjing, China). The reference substances of **5**, **11**, and **18** were purchased from Chengdu Purify-Biotechology Co., Ltd. (Chengdu, China). The reference substance of **6** was purchased from Nanjing Spring & Autumn Biological Engineering Co., Ltd.(Nanjing, China). The reference substance of **14** was purchased from Sigma-Aldrich (St. Louis, MO, USA). The reference substance of **16** was purchased from Chengdu Chroma-Biotechnology Co., Ltd. (Chengdu, China). The purities of all above reference substances were more than 98% determined by HPLC analysis.

### 2.2. Plant Materials

The EC (S1–S3), EF (S4–S6), and EFM (S7–S9) samples were collected from Lueyang City, Shaanxi Province (105°42′53″ N, 33°23′6″ E) in the traditional harvest time and dried at source area. The samples were authenticated by Prof. Xunhong Liu of the Nanjing University of Chinese Medicine, and the voucher specimens were deposited at the Herbarium in School of Pharmacy, Nanjing University of Chinese Medicine, China.

### 2.3. Preparation of Standard Solution

A mixed standard stock solution containing 21 reference substances was prepared by dissolving them in methanol and their concentrations were as follows: **1**, 7.60 μg/mL; **2**, 195.00 μg/mL; **3**, 8.32 μg/mL; **4**, 127.20 μg/mL; **5**, 9.80 μg/mL; **6**, 12.52 μg/mL; **7**, 19.44 μg/mL; **8**, 476.00 μg/mL; **9**, 75.00 μg/mL; **10**, 40.20 μg/mL; **11**, 118.80 μg/mL; **12**, 11.92 μg/mL; **13**, 67.70 μg/mL; **14**, 58.40 μg/mL; **15**, 33.50 μg/mL; **16**, 11.80 μg/mL; **17**, 13.44 μg/mL; **18**, 44.80 μg/mL; **19**, 13.00 μg/mL; **20**, 7.74 μg/mL; **21**, 62.95 μg/mL. The mixed standard stock solution was then diluted with methanol to a series of appropriate concentrations for construction of the calibration curves. All of the solutions were stored at 4 °C and filtered through the 0.22 μm membranes (Jinteng laboratory equipment Co., Ltd., Tianjin, China) prior to injection for LC-MS analysis.

### 2.4. Preparation of Sample Solutions

0.6 g of sample powder, after passing through a 40 mesh sieve, was weighed accurately and ultrasonically extracted with 30 mL 50% (*v*/*v*) methanol for 20 min and cooled at room temperature, then 50% (*v*/*v*) methanol was added to compensate for the lost weight. The resultant solution was subsequently centrifuged at 12,000 rpm for 10 min and the supernatant was stored at 4 °C and filtered through a 0.22 μm membrane (Jinteng laboratory equipment Co., Ltd., Tianjin, China) prior to injection for LC-MS analysis.

### 2.5. Chromatographic and Mass Spectrometric Conditions

UFLC was performed by using an UFLC-20ADXR system (Shimadzu, Kyoto, Japan). All separations were carried out on a Synergi™ Hydro-RP 100Å column (100 mm × 2.0 mm, 2.5 μm). The mobile phase is consisted of A (0.1% aqueous formic acid) and B (acetonitrile with 0.1% formic acid) with a gradient elution as follows: 0–2 min, 10%–40% B; 2–4 min, 40%–55% B; 4–7 min, 55%–95% B; 7–11 min, 95% B; 11–12 min, 95%–10% B. The re-equilibration time was 3 min and the flow rate of the mobile phase was 0.3 mL/min. The column temperature was maintained at 30 °C and the injection volume was 1 μL.

Mass spectrometry was performed using an API 5500 triple quadrupole mass spectrometer (AB Sciex, Framingham, MA, USA) equipped with an electrospray ionization (ESI) source operating in both positive and negative ion modes. The parameters in the source were set as follows: GS1 flow, 65 L/min; GS2 flow, 65 L/min; CUR flow, 30 L/min; gas temperature, 650 °C; pressures of nebulizer of MS, 4500 V (positive) and −4500 V (negative). All MS data were acquired using the Analyst 1.6.2 software to ensure mass accuracy and reproducibility.

### 2.6. Validation of The Method

The proposed method was validated according to the International Conference on Harmonisation (ICH) guidelines Q2 (R1) [21]. The principal parameters studied were linearity and range, limits of detection and quantification (LOD and LOQ), precision, repeatability, solution stability, and accuracy.

#### 2.6.1. Linearity and Range, LOD, and LOQ

The standard solution containing 21 compounds was prepared and diluted with methanol to appropriate concentrations for the construction of calibration curves. Calibration curves were developed by plotting the peak areas versus the corresponding concentrations of each analyte. The correlation coefficient, slope, and y-intercept were calculated for each replicate with acceptance criteria of a correlation coefficient r ≥ 0.99. 

The LOD and LOQ of 21 compounds were measured at signal-to-noise ratios of 3 and 10, respectively. 

#### 2.6.2. Precision, Repeatability, Solution Stability, and Accuracy

Intra- and inter-day variations were used to evaluate the precision of the established method. The relative standard deviation (RSD) of the peak area was taken as a measure of precision. 

To confirm the repeatability, six different analytical sample solutions prepared from the same sample were analyzed. The RSD of the peak area was taken as a measure of repeatability.

A stability test was further performed to analyze the variations in the sample solutions at 0, 2, 4, 8, 12, and 24 h, respectively. 

A recovery test was used to evaluate the accuracy of the established method. The test was performed by adding the corresponding marker constituents at low (80% of the known amounts), medium (same as the known amounts), and high (120% of the known amounts) levels to the EC sample which had been analyzed previously. The mixture was extracted and analyzed using the aforementioned method in triplicate.

### 2.7. Principal Component Analysis (PCA)

PCA is an unsupervised pattern recognition method used for analyzing, classifying, and reducing the dimensionality of numerical datasets in a multivariate problem [22], and it has been widely used for the quality control of herbal medicines [23,24,25]. Data of the contents of 21 compounds in EC, EF, and EFM samples were listed in an Excel file. PCA was used to evaluate the variations of the three different parts of *Eucommia ulmoides* according to the contents of the 21 bioactive compounds analyzed in this study using Simca-P 13.0 (version 13.0, Umetrics AB, Umea, Sweden) software. The data which shown in Table 3 was imported into Simca-P 13.0 and centralized by Simca-P 13.0, and a data matrix after centralization is obtained which was shown in Table S1. When the contents of investigated compounds were below the quantitation limit or not detected in the samples, the values of such elements were considered to be 0. 

## 3. Results and Discussion

### 3.1. Optimization of Extraction Conditions

In order to achieve an efficient extraction of bioactive constituents in samples, orthogonal test was employed to investigate these key factors, including methanol concentration (30% (*v/v*) methanol, 50% (*v/v*) methanol, and 80% (*v/v*) methanol), sample-solvent ratio (1:30, 1:50, and 1:80 (*w*/*v*)), and ultrasonic time (10 min, 20 min, and 40 min). Finally, the optimum sample extraction condition was achieved by ultrasonic extraction with a 1:50 ratio of 50% methanol for 20 min. All of the samples were extracted at room temperature.

### 3.2. Optimization of UFLC Conditions

To obtain the optimum elution conditions, four types of reverse phase columns including Thermo Acclaim™ RSLC 120 C_18_ (100 mm × 2. 1 mm,2.2 μm), Inertsil ODS-3 column (250 mm × 4.6 mm, 5 μm), ZOBRX SB-C_18_ (250 mm × 4.6 mm, 5 μm), and Synergi™ Hydro-RP 100Å column (100 mm × 2.0 mm, 2.5 μm) were evaluated; as a result, better adequate retention, selectivity, and peak shape were achieved on a Synergi™ Hydro-RP 100Å column (100 mm × 2.0 mm, 2.5 μm). Various UFLC parameters including a mobile phase modifier (methanol-water, acetonitrile-water, acetonitrile with 0.1% formic acid-0.1% aqueous formic acid), column temperature (25, 30, and 40 °C), and flow rate (0.2, 0.3, and 0.4 mL/min) were investigated. Satisfactory separation and better ionization could be achieved when the acetonitrile with 0.1% formic acid-0.1% aqueous formic acid was used as the mobile phase. Similarly, satisfactory resolution in shortest analysis time was obtained at a flow rate of 0.3 mL/min and at 30 °C column temperature. Hence, acetonitrile with 0.1% formic acid-0.1% aqueous formic acid at the flow rate of 0.3 mL/min and at 30 °C on Synergi™ Hydro-RP 100Å column (100 mm × 2.0 mm, 2.5 μm) was finally selected and applied. 

### 3.3. Optimization of MS Conditions

Preliminary experiments were conducted with the purpose of finding the best instrumental conditions. The individual solutions of all standard compounds (100 ng/mL in methanol) were injected into the ESI source in the positive and negative ion modes. After trial and error inspection, other compounds have a good condition in the negative ion mode, while syringin has a good condition in the positive ion mode. Most abundance fragment ions were selected as MRM transition from MS/MS spectrum, and the highest sensitivity was obtained at a certain value of fragmentor and collision energy (CE). The optimum values for each condition of 21 compounds were summarized in Table 1. 

### 3.4. UFLC Method Validation

#### 3.4.1. Linearity and Range, LOD, and LOQ

The calibration curve for each compound was obtained in duplicate with at least six appropriate concentrations. The correlation coefficients of all target components exceeded 0.9991 with good linearity. The LODs and LOQs of 21 compounds were measured at signal-to-noise ratios of 3 and 10 and the ranges were 0.32–4.76 ng/mL and 1.15–16.66 ng/mL, respectively. The results were shown in Table 2.

#### 3.4.2. Precision, Repeatability, Solution Stability, and Accuracy

For the intra-day variability test, the mixed standard solutions were analyzed for six replicates within a day; for the inter-day variability test, the solutions were examined for three consecutive days. The relative standard deviation (RSD) was taken as a measure of precision. The RSD values of intra- and inter-day variations of 21 compounds were in the range of 1.09%–3.45% and 2.10%–3.76%, respectively. The results were shown in Table 2.

The satisfactory repeatabilities presented as RSD values were in the range of 1.02% to 3.68%. The results were shown in Table 2.

The solution stabilities presented as RSD values were less than 3.85%, indicating the sample solution was stable when stored at room temperature for 24 h. The results were shown in Table 2.

The overall recoveries lay between 95.83% and 103.30%, with RSD values between 1.03% and 3.98%. The results were shown in Table 2.

### 3.5. Sample Analysis

**Figure 1:**
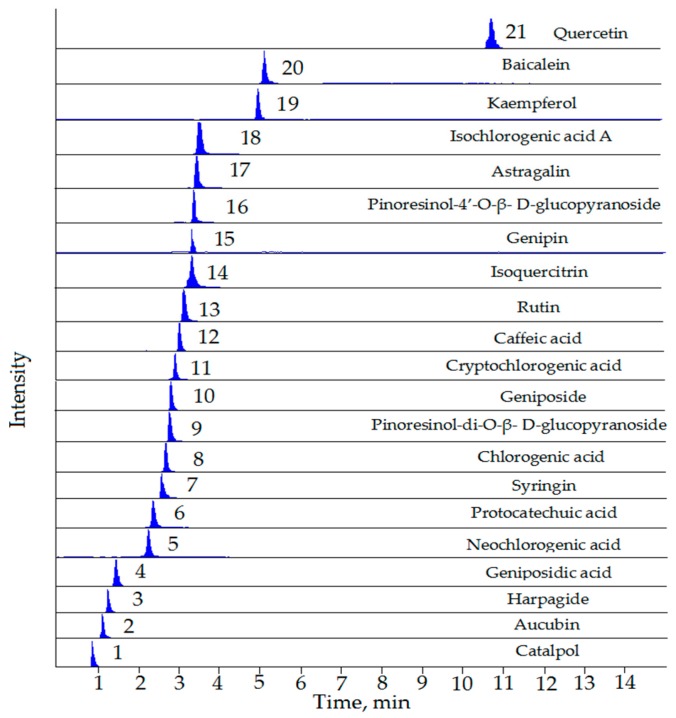
Representative extract ions chromatograms (XIC) of multiple-reaction monitoring (MRM) chromatograms of the 21 investigated compounds.

The developed UFLC-QTRAP-MS/MS method was subsequently applied to the comprehensive quality evaluation of EC, EF, and EFM samples. The contents of 21 compounds were simultaneously determinated in this study. In 10 metabolites isolated from EU by the authors, which were determinated in the study by Chai et al. [17], we determinated 8 metabolites. The contents of wogonin and oroxylin A in the samples were too low to meet the linear range. Additionally, the standards of licoagroside F and syringaresinol di-*O*-β-d-glucopyranoside were not obtained. Typical MRM chromatograms were shown in Figure 1, and the results of the quantitative determination of 21 compounds from these samples were summarized in Table 3. The results were reported as mean ± SD. 

By comparing the amounts, it was found that the constituents of EC, EF, and EFM samples were quite different. The total contents of 21 constituents varied from 5132.65 μg/g to 34,200.10 μg/g. The total contents of each type of constituent were also calculated; two lignans ranged from 16.37 μg/g to 1497.00 μg/g, 6 iridoids ranged from 172.48 μg/g to 10,187.34 μg/g, 6 penylpropanoids ranged from 2128.32 μg/g to 21,421.96 μg/g, 6 flavonoids ranged from 0.21 μg/g to 4944.46 μg/g, and one phenol ranged from 4.85 μg/g to 262.80 μg/g. The results indicated that the contents of the 21 compounds were obviously different in the three different parts of EC.

**Figure 2:**
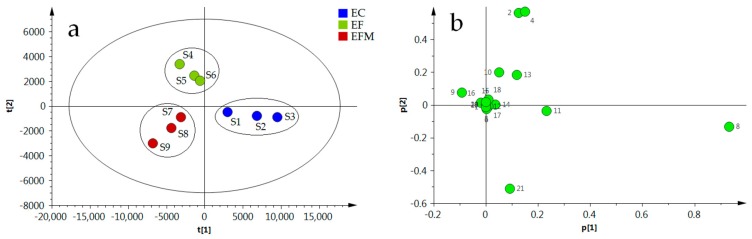
PCA score scatter plot (**a**) and PCA loading plot (**b**) of different parts of EU.

To evaluate the differences in chemical composition in different medicinal parts of EC, unsupervised PCA was performed. The contents of 21 compounds were set as variables, while 9 samples were set as observations. The score scatter plot and the loading scatter plot were displayed in Figure 2. The first and second principal components described 82.7% and 13.1% of the variability in the original observations, respectively. Consequently, the first two principal components accounted for 95.8% of the total variance. The PCA score scatter plot (2a) showed that the samples of EC, EF, and EFM clustered in three small regions, which means the chemical compositions in EC, EF, and EFM were significantly different. In the loading scatter plot (2b), it can be observed that different variables made different contributions to sample differentiation; the points located near to each other indicated the contents of principal components were similar. The p1 and p2 values of all points of the loading scatter plot are shown in Table S2. It can be seen that the chemical markers responsible for the cluster formation are mainly compounds **2** (aucubin), **4** (geniposidic acid), **8** (chlorogenic acid), **9** (pinoresinol-di-*O*-β-d-glucopyranoside), **10** (geniposide), **11** (cryptochlorogenic acid), **13** (rutin), and **21** (quercetin). As can be seen in the loading scatter plot, compound **8** (p1: 0.933, p2: −0.133), compound **11** (p1: 0.233, p2: −0.038), compound **4** (p1: 0.150, p2: 0.568), compound **2** (p1: 0.126, p2: 0.561), compound **21** (p1: 0.094, p2: −0.512), compound **13** (p1: 0.119, p2: 0.183), compound **10** (p1: 0.052, p2: 0.198), and compound **9** (p1: −0.092, p2: 0.074) were far from the center, so these compounds were believed to have significant relationship with sample classifications. The PCA results indicated the chemical composition of EC, EF, and EFM were evidently different, and the compounds **2**, **4**, **8**, **9**, **10**, **11**, **13**, and **21** had significant relationship with sample classifications.

## 4. Conclusions

In this study, an efficient and sensitive UFLC-QTRAP-MS/MS method has been developed and validated for the simultaneous determination of a total of 21 constituents, including two lignans, 6 iridoids, 6 penylpropanoids, 6 flavonoids, and one phenol in EU samples. The validated method was successfully applied to quantify 21 bioactive constituents in EC, EF, and EFM. PCA was performed to evaluate and classify the samples according to the contents of these 21 constituents. All of the results demonstrated that the chemical compositions in EC, EF, and EFM were significantly different, and the differential constituents (i.e., aucubin, geniposidic acid, chlorogenic acid, pinoresinol-di-*O*-β-d-glucopyranoside, geniposide, cryptochlorogenic acid, rutin, and quercetin) had a significant relationship with the sample classifications. This research will provide the basic foundation for revealing the laws of metabolite accumulation in EC, EF, and EFM from the same origin.

## Figures and Tables

**Table 1:** Retention time, and related MS data of the target compounds.

No.	Compounds	RT (min)	Precursor Ion [M − H]^−^ (*m*/*z*)	Product Ion (*m*/*z*)	Fragmentor (V)	CE (eV)
1	Catalpol	0.90	361.2	169.0	−60	−18
2	Aucubin	1.18	345.1	183.0	−50	−22
3	Harpagide	1.26	363.0	201.0	−135	−18
4	Geniposidic acid	1.47	373.1	123.0	−130	−26
5	Neochlorogenic acid	2.24	353.0	191.0	−95	−20
6	Protocatechuic acid	2.37	152.9	109.0	−85	−16
7 *	Syringin	2.59	395.2	232.1	136	31
8	Chlorogenic acid	2.71	353.0	191.0	−95	−20
9	Pinoresinol-di-*O*-β- d-glucopyranoside	2.79	681.2	357.3	−155	−34
10	Geniposide	2.82	387.1	225.0	−180	−12
11	Cryptochlorogenic acid	2.89	353.0	191.0	−95	−20
12	Caffeic acid	3.05	179.0	134.6	−125	−20
13	Rutin	3.15	609.1	300.0	−245	−46
14	Isoquercitrin	3.31	463.0	299.9	−180	−36
15	Genipin	3.33	225.0	101.1	−30	−16
16	Pinoresinol-4’-*O*-β- d-glucopyranoside	3.36	519.2	357.1	−160	−22
17	Astragalin	3.45	447.1	283.9	−100	−36
18	Isochlorogenic acid A	3.53	515.1	191.0	−145	−38
19	Kaempferol	4.98	285.0	116.9	−180	−48
20	Baicalein	5.15	269.0	139.0	−130	−40
21	Quercetin	10.78	301.1	255.1	−45	−10

* The compound was detected in positive mode as Na-adduct.

**Table 2:** Regression equation, LOD and LOQ, precision, repeatability, stability, and recovery of 21 investigated compounds.

No.	Compounds	Regression Equation	*r*^2^	Liner Range (μg/mL)	LOD (ng/mL)	LOQ (ng/mL)	Precision RSD (%)		Repeatability RSD (%) (*n* = 6)	Stability RSD (%)	Recovery (%) (*n* = 3)					
							Intar-Day (*n* = 6)	Inter-Day (*n* = 6)			Low		Medium		High	
											Mean	RSD	Mean	RSD	Mean	RSD
1	Catalpol	Y = 20.5X + 888	0.9997	0.015–7.600	1.52	5.32	2.22	3.70	3.68	1.44	98.67	1.62	98.34	2.74	99.26	3.35
2	Aucubin	Y = 9.05X + 13,800	0.9997	0.130–195.000	3.90	13.65	3.45	2.10	2.87	1.16	99.00	2.27	99.16	1.78	98.77	2.10
3	Harpagide	Y = 211X + 2990	0.9999	0.017–8.320	1.44	3.98	1.41	2.93	3.65	3.73	97.62	3.14	97.47	3.98	97.56	3.68
4	Geniposidic acid	Y = 1060X + 48,400	0.9999	0.025–127.200	1.27	4.45	2.15	2.62	2.09	3.85	99.91	3.08	98.43	1.76	97.56	2.55
5	Neochlorogenic acid	Y = 2130X + 26,000	0.9999	0.020–9.800	1.96	6.86	1.45	2.40	2.05	1.34	99.77	3.67	96.45	3.81	98.01	2.15
6	Protocatechuic acid	Y = 3210X + 171,000	0.9996	0.013–12.520	1.25	4.38	2.08	3.39	3.23	1.86	95.83	3.09	99.05	3.40	97.01	3.55
7	Syringin	Y = 115X + 7860	0.9997	0.019–19.440	1.94	6.80	2.96	2.23	3.56	1.24	98.38	2.29	100.16	2.82	98.81	2.82
8	Chlorogenic acid	Y = 170X + 176,000	0.9996	0.238–476.000	4.76	16.66	1.88	2.61	1.57	2.76	98.20	2.64	98.24	2.19	97.59	2.85
9	Pinoresinol-di-*O*-β- d-glucopyranoside	Y = 423X + 15,200	0.9998	0.150–75.000	3.75	13.13	1.09	3.07	2.83	3.20	98.29	1.20	98.01	2.92	96.72	3.88
10	Geniposide	Y = 48.3X + 36,700	0.9991	0.080–40.200	1.47	4.94	1.20	2.16	1.45	2.64	98.20	3.85	96.45	1.17	96.99	3.52
11	Cryptochlorogenic acid	Y = 350.5X + 506,010	0.9996	0.119–118.800	2.97	10.40	2.04	3.57	1.94	1.63	96.83	2.08	96.13	3.31	98.34	3.19
12	Caffeic acid	Y = 2540X + 114,000	0.9998	0.024–11.920	1.19	4.17	1.87	2.91	2.74	3.84	98.38	3.68	96.95	3.37	100.43	3.07
13	Rutin	Y = 652X + 238,000	0.9999	0.135–67.700	0.60	1.99	1.39	3.58	1.54	3.57	98.15	3.82	97.28	3.45	95.48	2.96
14	Isoquercitrin	Y = 2640X + 216,000	0.9995	0.117–58.400	0.89	3.02	2.15	3.29	2.09	2.54	98.16	3.55	96.59	2.08	102.03	3.16
15	Genipin	Y = 953X + 68,600	0.9991	0.034–33.500	0.67	2.35	3.26	3.14	2.68	3.30	98.41	2.29	96.34	3.44	98.09	3.55
16	Pinoresinol-4’-*O*-β- d-glucopyranoside	Y = 0.89X +116	0.9991	0.022–11.180	1.12	3.91	2.70	2.40	3.60	2.99	102.35	3.29	97.18	2.95	97.43	2.13
17	Astragalin	Y = 3050X + 130,000	0.9999	0.027–13.440	0.98	2.96	2.07	3.04	2.55	2.80	99.89	3.87	98.52	1.99	96.71	2.18
18	Isochlorogenic acid A	Y = 1300X + 149,000	1.0000	0.090–44.800	2.24	7.84	2.99	3.07	3.31	3.59	97.34	3.49	97.69	2.58	97.07	2.98
19	Kaempferol	Y = 429X + 1010	1.0000	0.026–13.000	2.36	5.90	1.87	2.16	3.77	3.36	96.13	3.15	96.85	2.93	97.92	2.04
20	Baicalein	Y = 531X − 1080	0.9991	0.0015–7.740	0.32	1.15	2.68	3.08	3.04	3.57	97.62	3.52	103.30	2.38	98.64	3.38
21	Quercetin	Y = 2.46X + 123,000	0.9991	0.126–62.950	1.45	4.69	2.14	3.76	1.02	3.38	95.91	3.41	98.65	3.73	97.45	2.03

**Table 3:** Contents of 21 compounds in EC, EF, and EFM (μg/g, mean ± SD, *n* = 3).

No.	Compounds	EC			EF			EFM		
		S1	S2	S3	S4	S5	S6	S7	S8	S9
1	Catalpol	26.33 ± 2.77	14.45 ± 1.82	18.80 ± 1.23	54.78 ± 3.60	91.33 ± 4.54	135.17 ± 4.80	18.80 ± 1.47	15.89 ± 0.60	21.07 ± 0.91
2	Aucubin	351.33 ± 34.59	557.00 ± 12.53	895.00 ± 13.23	27.70 ± 3.34	45.02 ± 3.11	17.71 ± 2.26	84.00 ± 7.21	2378.33 ± 12.58	4663.33 ± 132.04
3	Harpagide	2.43 ± 0.57	1.23 ± 0.05	4.15 ± 0.28	1.65 ± 0.04	2.06 ± 0.06	1.14 ± 0.10	2.66 ± 0.32	1.30 ± 0.05	-
4	Geniposidic acid	1630.00 ± 5.00	1040.00 ± 36.06	393.67 ± 7.51	129.17 ± 2.52	32.22 ± 2.06	67.11 ± 4.43	3285.00 ± 97.60	3541.67 ± 68.25	3628.33 ± 46.46
5	Neochlorogenic acid	31.52 ± 0.87	22.18 ± 0.28	11.20 ± 0.30	139.40 ± 0.85	83.80 ± 1.75	67.33 ± 5.69	28.94 ± 0.45	28.98 ± 0.81	30.21 ± 1.03
6	Protocatechuic acid	13.87 ± 0.39	4.85 ± 0.37	8.32 ± 0.69	43.25 ± 1.38	262.80 ± 8.78	153.17 ± 10.60	37.27 ± 1.81	31.78 ± 0.78	26.10 ± 0.48
7	Syringin	1.33 ± 0.20	5.40 ± 0.22	7.98 ± 0.50	37.37 ± 2.13	1.25 ± 0.26	6.29 ± 0.50	30.63 ± 0.74	25.12 ± 0.83	27.73 ± 1.52
8	Chlorogenic acid	8030.00 ± 62.45	4276.67 ± 102.14	1641.33 ± 116.57	14,086.67 ± 160.42	12,446.67 ± 291.43	11,593.33 ± 30.55	13,410.00 ± 284.78	14,543.33 ± 695.15	16,710.00 ± 608.52
9	Pinoresinol-di-*O*-β-d-glucopyranoside	888.33 ± 32.15	1295.00 ± 30.00	1125.00 ± 52.20	9.07 ± 0.55	15.91 ± 0.70	21.72 ± 1.01	16.88 ± 0.75	26.92 ± 2.54	74.17 ± 3.88
10	Geniposide	13.68 ± 0.98	231.33 ± 4.25	148.13 ± 6.79	-	-	-	111.83 ± 0.76	578.33 ± 20.82	1803.33 ± 35.12
11	Cryptochlorogenic acid	2013.33 ± 20.82	1073.33 ± 30.55	406.00 ± 27.22	3553.33 ± 70.24	3100.00 ± 70.00	2963.33 ± 118.46	3343.33 ± 75.72	3550.00 ± 90.00	4230.00 ± 115.33
12	Caffeic acid	31.32 ± 0.31	20.17 ± 0.81	14.79 ± 0.56	29.05 ± 1.13	182.50 ± 4.58	38.03 ± 1.95	51.28 ± 1.63	37.52 ± 1.93	20.62 ± 0.81
13	Rutin	-	-	-	541.00 ± 28.16	88.67 ± 2.52	343.00 ± 26.51	2561.67 ± 25.17	1509.33 ± 80.60	1685.00 ± 10.00
14	Isoquercitrin	-	-	-	475.00 ± 7.55	108.33 ± 1.53	369.67 ± 20.51	395.33 ± 11.56	441.00 ± 22.11	507.00 ± 14.73
15	Genipin	10.18 ± 0.83	92.83 ± 0.29	104.33 ± 6.03	11.50 ± 0.92	1.85 ± 0.18	18.22 ± 1.18	407.50 ± 15.55	36.38 ± 3.59	71.28 ± 4.08
16	Pinoresinol-4′-*O*-β-d-glucopyranoside	138.33 ± 1.26	202.00 ± 2.65	306.50 ± 17.26	7.30 ± 0.23	2.28 ± 0.01	6.19 ± 0.05	12.49 ± 0.94	11.22 ± 0.23	13.33 ± 0.45
17	Astragalin	-	-	-	165.50 ± 4.27	47.65 ± 0.23	160.73 ± 13.51	106.53 ± 4.00	116.33 ± 5.51	118.50 ± 9.00
18	Isochlorogenic acid A	100.17 ± 5.13	62.40 ± 0.85	47.02 ± 3.06	117.33 ± 2.52	11.07 ± 0.21	32.90 ± 0.90	95.17 ± 4.86	83.83 ± 1.04	403.40 ± 9.56
19	Kaempferol	-	-	-	22.13 ± 0.57	31.08 ± 0.58	20.07 ± 1.31	19.32 ± 1.09	5.99 ± 0.29	6.06 ± 0.23
20	Baicalein	0.21 ± 0.04	0.77 ± 0.09	0.43 ± 0.04	0.16 ± 0.01	0.15 ± 0.00	0.14 ± 0.01	0.14 ± 0.02	0.12 ± 0.00	0.14 ± 0.02
21	Quercetin	-	-	-	3740.67 ± 168.84	2085.00 ± 5.00	1651.67 ± 106.93	708.33 ± 5.77	637.83 ± 15.61	160.50 ± 5.29

## Supplementary Materials

The following are available online. Figure S1: Chemical structures of 21 reference substances, Table S1: The data matrix applying PCA, Table S2: The p1 and p2 values of the points in PCA loading plot.
