# Supplementary material for: Comparison of Multiple Bioactive Constituents in Different Parts of Eucommia ulmoides Based on UFLC-QTRAP-MS/MS Combined with PCA
Source: Molecules. 2018 Mar 13;23(3):643. doi: 10.3390/molecules23030643 (PMC6017739; doi:10.3390/molecules23030643)
Supplement: Supplementary file 1 [file molecules-23-00643-s001.zip › molecules-269412-supplementary.pdf]

Supplementary Material

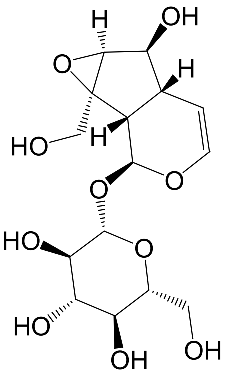

Catalpol (**1**)

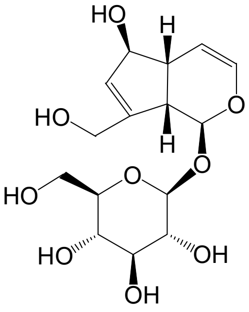

Aucubin (2)

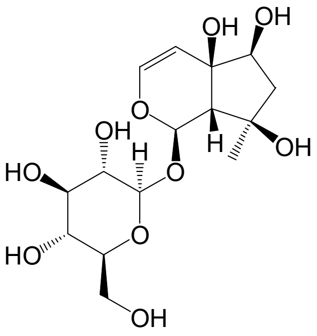

Harpagide (**3**)

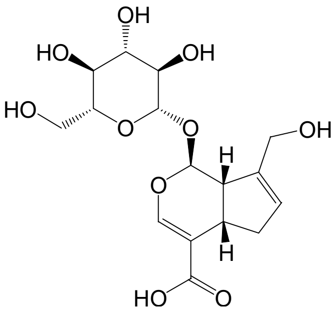

Geniposidic acid (**4**)

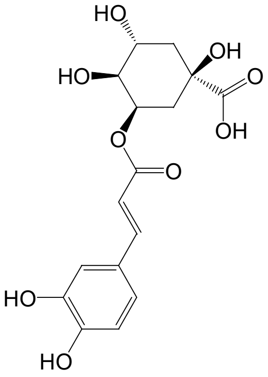

Neochlorogenic acid (**5**)

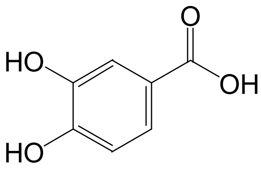

Protocatechuic acid (**6**)

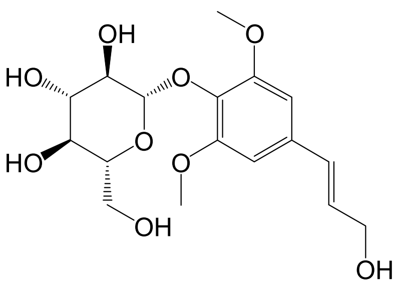

Syringin (**7**)

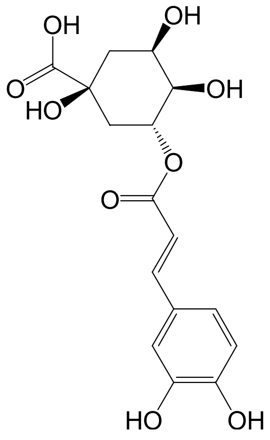

Chlorogenic acid (**8**)

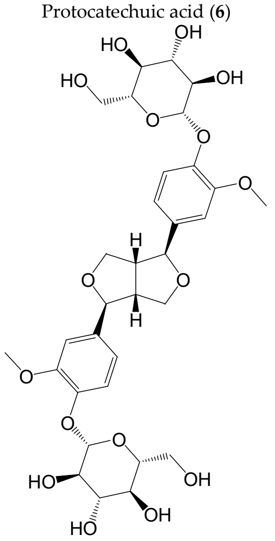

Pinoresinol-di-*O*-β-D-glucopyranoside (**9**)

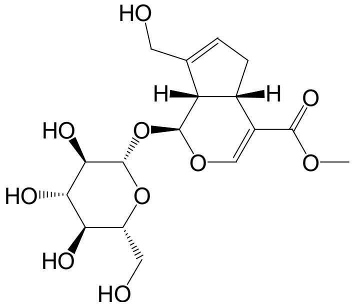
Geniposide (**10**)

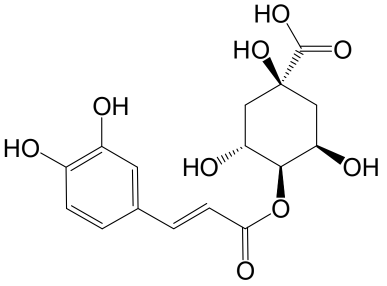
Cryptochlorogenic acid (**11**)

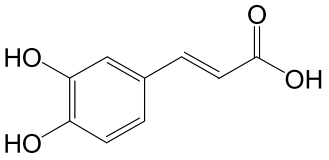
Caffeic acid (**12**)

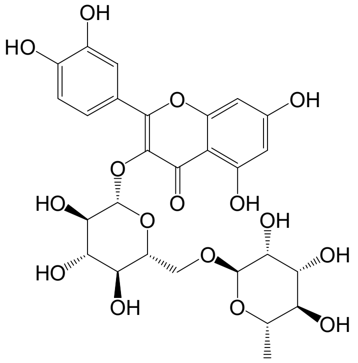
Rutin (**13**)

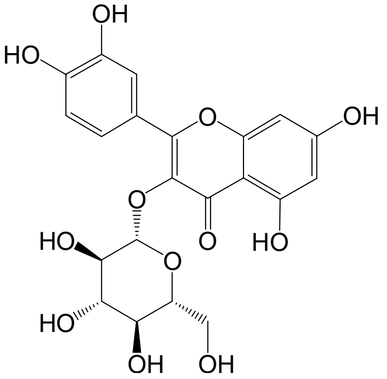
Isoquercitrin (**14**)

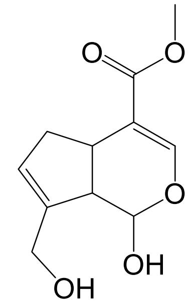
Genipin (**15**)

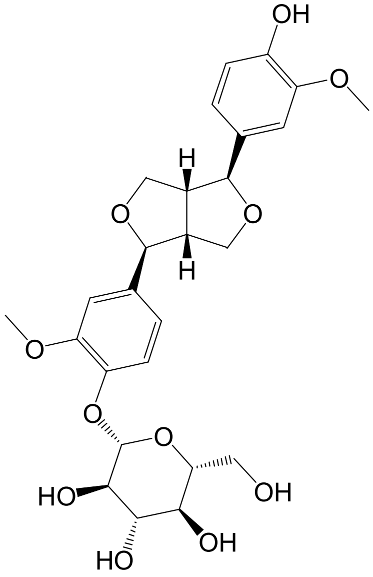
Pinoresinol-4′-*O*-β-D-glucopyranoside (**16**)

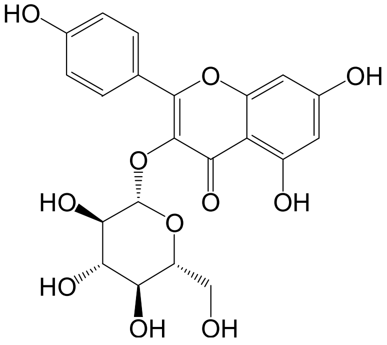
Astragalin (**17**)

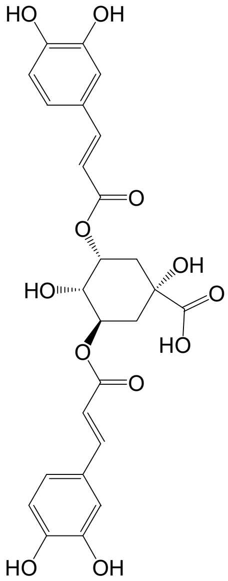
Isochlorogenic acid A (**18**)

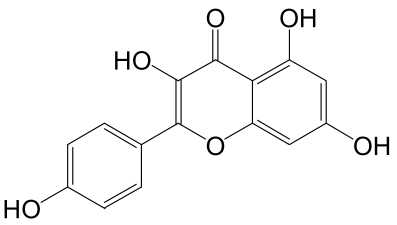
Kaempferol (**19**)

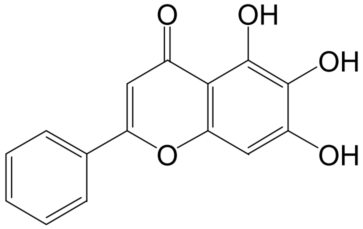
Baicalein (**20**)

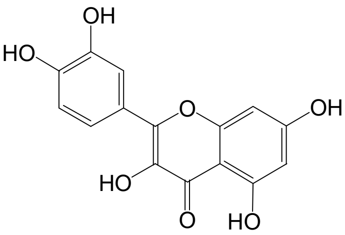
Quercetin (**21**)

Figure S1. Chemical structures of 21 reference substances.

Table S1. The data matrix applying PCA

No.	S1	S2	S3	S4	S5	S6	S7	S8	S9
1	-17.3719	-29.2485	-24.8985	11.0848	47.6348	91.4682	-24.8985	-27.8052	-25.9652
2	-650.861	-445.194	-107.194	-974.494	-956.844	-984.494	-918.194	1376.14	3661.14
3	0.736815	-0.789519	1.45848	-0.0598519	0.370148	-0.551519	0.636815	-0.396519	-1.40485
4	106.241	-517.092	-1130.09	-1394.59	-1491.54	-1456.65	1761.24	2017.91	2104.57
5	-17.7685	-27.1019	-38.0852	90.1148	34.5148	18.0482	-20.3485	-20.3019	-19.0719
6	-50.7337	-59.747	-56.2837	-21.3504	198.2	88.5663	-27.3337	-32.817	-38.5004
7	-13.9817	-11.2467	-8.33667	22.055	-14.0067	-8.68833	8.65167	13.135	12.4183
8	-2718.67	-6472	-9107.33	3338	1698	844.666	2661.33	3794.67	5961.33
9	502.445	909.112	739.112	-376.822	-369.975	-364.172	-369.005	-358.972	-311.722
10	-307.056	-89.4056	-172.606	-320.739	-320.739	-320.739	-208.906	257.594	1482.59
11	-679.185	-1619.19	-2286.52	860.815	407.482	270.815	650.815	857.482	1537.48
12	-15.7878	-27.9344	-32.6511	-18.0544	135.396	-9.07111	4.17889	-9.58778	-26.4878
13	-747.63	-747.63	-747.63	-206.63	-658.963	-404.63	1814.04	761.704	937.37
14	-255.148	-255.148	-255.148	219.852	-146.815	114.519	140.185	185.852	251.852
15	-73.2702	9.37982	20.8798	-71.9535	-81.6052	-68.2369	324.046	-47.0702	-12.1702
16	60.5952	124.262	228.762	-70.4381	-75.4581	-71.5448	-65.2515	-66.5215	-64.4048
17	-79.4722	-79.4722	-79.4722	86.0278	-31.8222	81.2611	27.0611	36.8611	39.0278
18	-5.79074	-43.5574	-58.9407	11.3759	-94.8907	-72.7241	-10.7907	-22.1241	297.443
19	-11.6278	-11.6278	-11.6278	10.5056	19.4556	8.43889	7.68889	-5.63444	-5.57111
20	-0.0319593	0.530207	0.116374	-0.0806259	-0.0940926	-0.105126	-0.108126	-0.122026	-0.104626
21	-997.111	-997.111	-997.111	2743.56	1087.89	654.556	-288.778	-359.278	-846.611

Table S2. The p1 and p2 values of the points in PCA loading plot

No.	p1	p2
1	0.00114	-0.0146
2	0.126	0.561
3	-0.0000861	-0.0000922
4	0.150	0.569
5	0.00287	-0.0162
6	0.00368	-0.0246
7	0.00177	0.000590
8	0.933	-0.133
9	-0.0920	0.0740
10	0.0521	0.198
11	0.234	-0.0383
12	0.00197	-0.0111
13	0.119	0.183
14	0.0363	0.00100
15	0.00112	0.0168
16	-0.0193	0.0150
17	0.00997	-0.00939
18	0.0114	0.0317
19	0.00108	-0.00403
20	-0.0000285	0.0000169
21	0.0935	-0.512
